# Supplementary figures and images for: Rod genesis driven by mafba in an nrl knockout zebrafish model with altered photoreceptor composition and progressive retinal degeneration
Source: PLoS Genet. 2022 Mar 4;18(3):e1009841. doi: 10.1371/journal.pgen.1009841 (PMC8926279; doi:10.1371/journal.pgen.1009841)

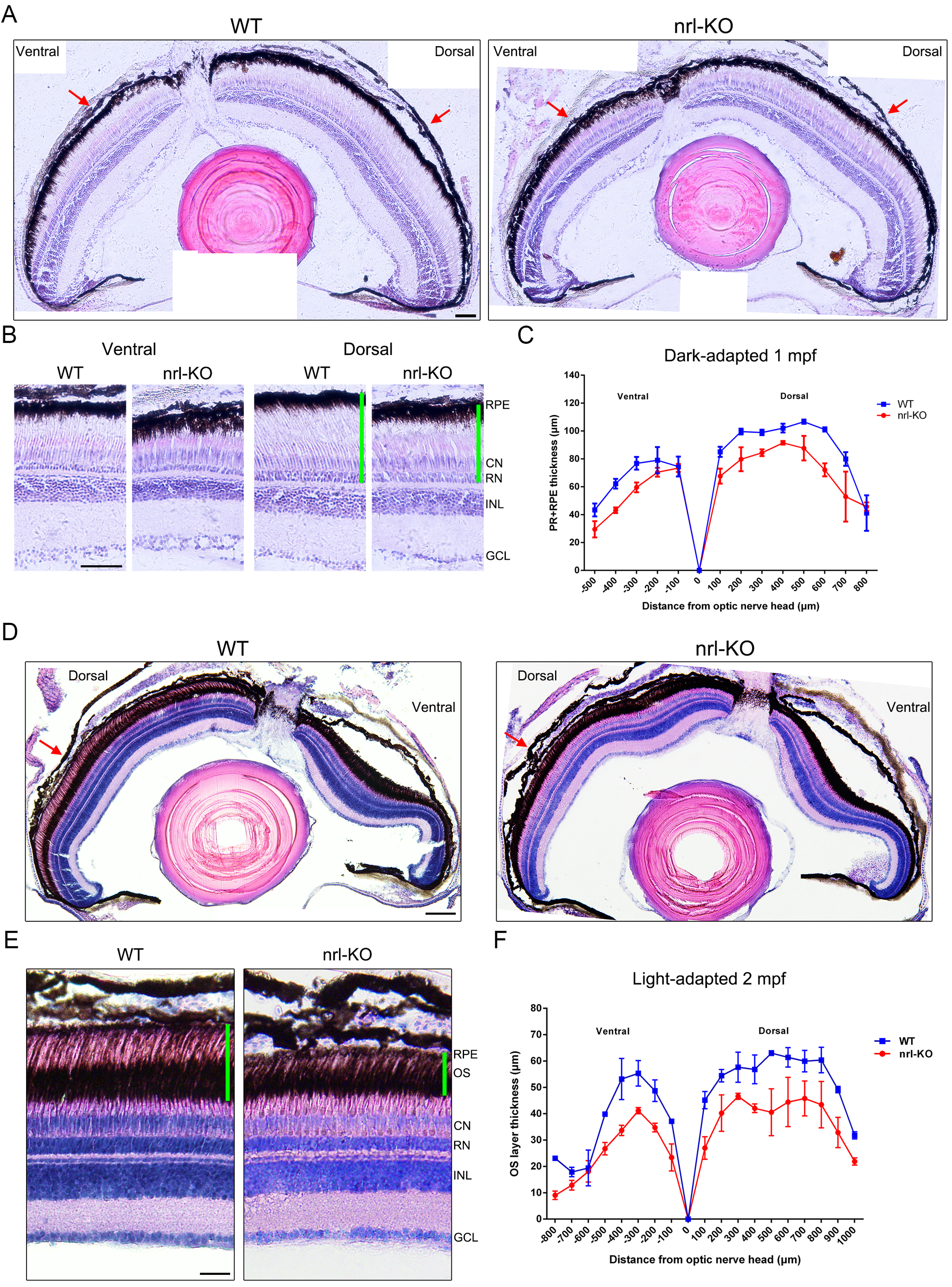

Supplement: S1 Fig — (A and D) HE staining of the retinal sections from the dark- and light-adapted WT and nrl-KO zebrafish at 1 mpf and 2 mpf, respectively. Scale bars: 50 μm in (A) and 100 μm in (D). (B and E) Enlarged images of the retinal regions indicated by red arrows in (A) and (D). The green vertical lines indicate the thicknesses of the PR+RPE layer in (B) and the OS layer in (E). Scale bars: 50 μm in (B) and 25 μm in (E). PR, photoreceptor; RPE, retinal pigment epithelium; OS, outer segment; IS, inner segment; ONL, outer nuclear layer; INL, inner nuclear layer; GCL, ganglion cell layer. (C and F) Quantitation the thicknesses of the PR+RPE layer in (B) and the OS layer in (D), as measured every 100 μm from the optic nerve to the edges of the ventral and dorsal retinas. The results are shown as mean with SD (n = 6 and n = 3, respectively). Blue lines, WT group; red lines, nrl-KO group. (TIF) [file pgen.1009841.s001.tif]

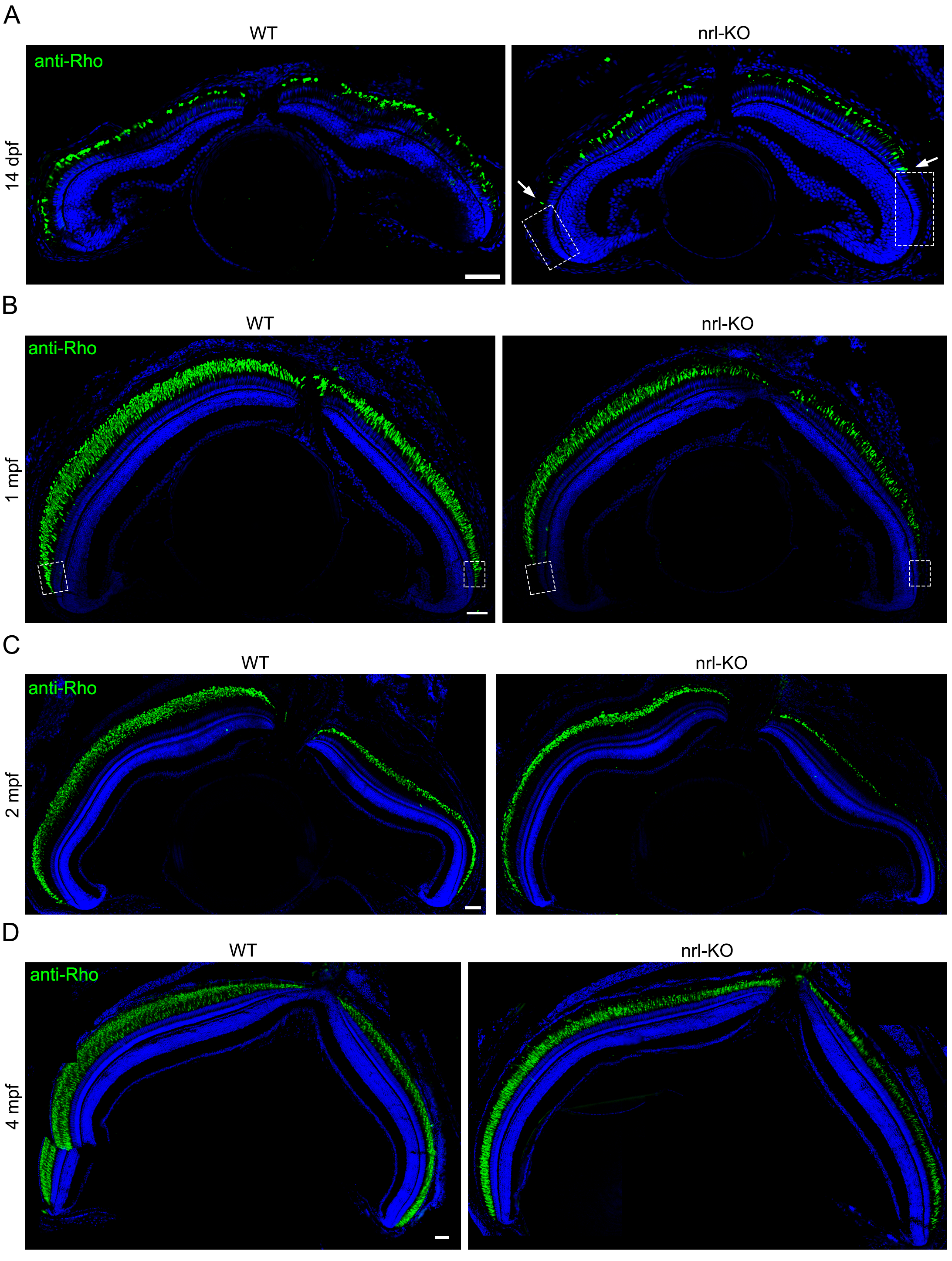

Supplement: S2 Fig — The rod outer segments were labeled with the anti-Rho antibody on retinal sections from the WT and nrl-KO zebrafish at 14 dpf (A), 1 mpf (B), 2 mpf (C), and 4 mpf (D). The regions nearby the ciliary marginal zone (labeled with boxes) showed no or weak fluorescence signal of rods in the nrl-KO retinas at 14 dpf and 1 mpf. Scale bars: 50 μm. (TIF) [file pgen.1009841.s002.tif]

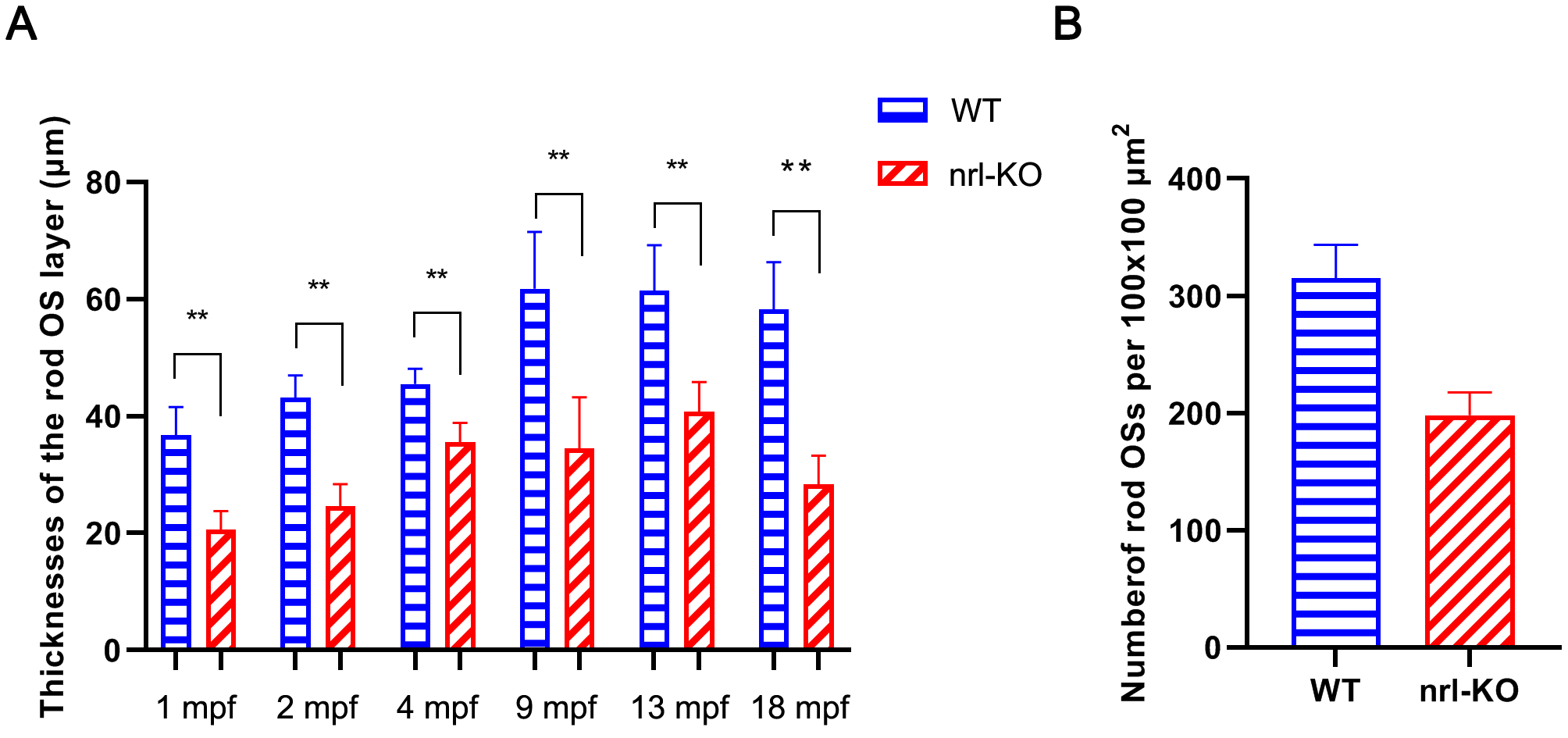

Supplement: S3 Fig — (A) The rod outer segments were visualized via immunostaining on retinal sections (see Figs 1D and S2) from 1 mpf to 18 mpf using the anti-Rho antibody. The thickness of the rod OS layer was measured in the dorsal-middle regions of the WT and nrl-KO retinas. The results are shown as mean with SD (n = 6). **, p < 0.01. (B) Immunostaining was performed on flattened whole-mount retinas to visualize rod outer segments at 2 mpf (see Fig 2). The density of the rod OSs (the number in a 100 μm x 100 μm region) were measured in the dorsal regions of the WT and nrl-KO retinas. The results are shown as mean with SD (n = 6). **, p < 0.01. (TIF) [file pgen.1009841.s003.tif]

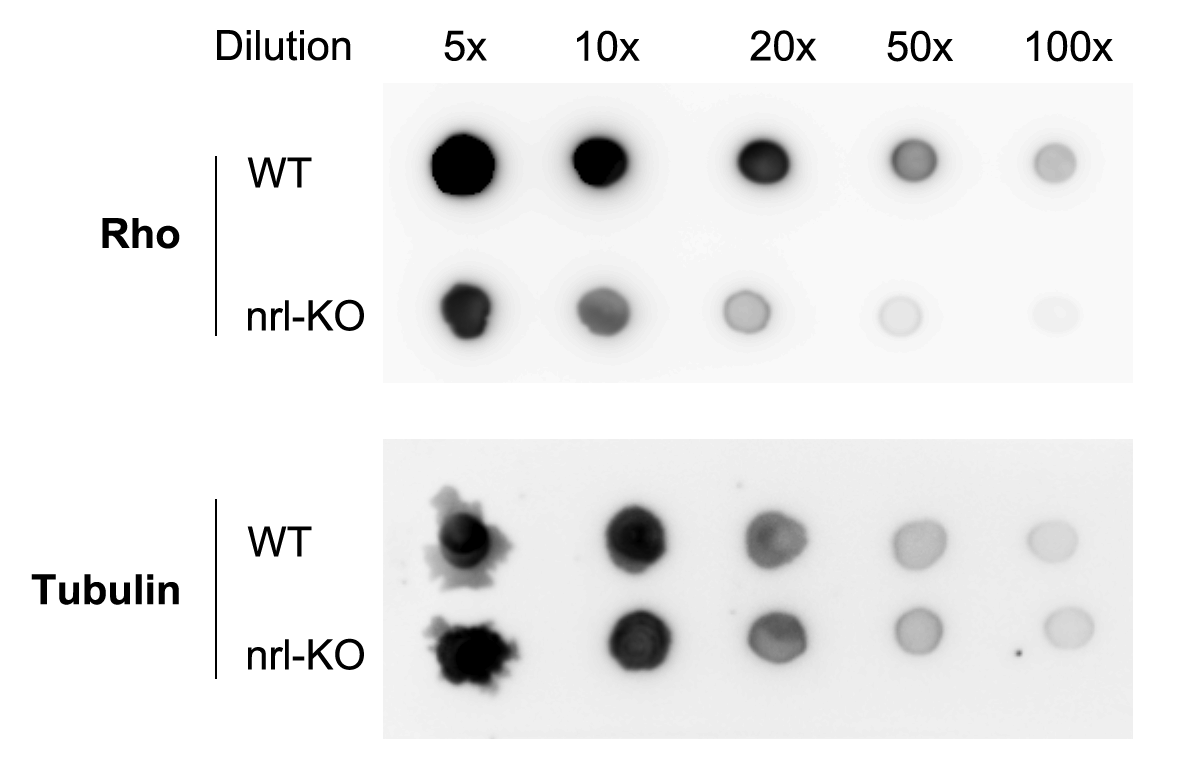

Supplement: S4 Fig — A series of gradient dilutions of retinal protein extracts from the WT and nrl-KO zebrafish at 9 mpf were spotted onto the NC membranes and immunoblotted with the anti-Rho antibody. Tubulin served as a loading control. By comparing the 10x dot in the nrl-KO group with the 20x and 50x dots in the WT group, Rho was estimated to be 2.5–5 fold downregulated. (TIF) [file pgen.1009841.s004.tif]

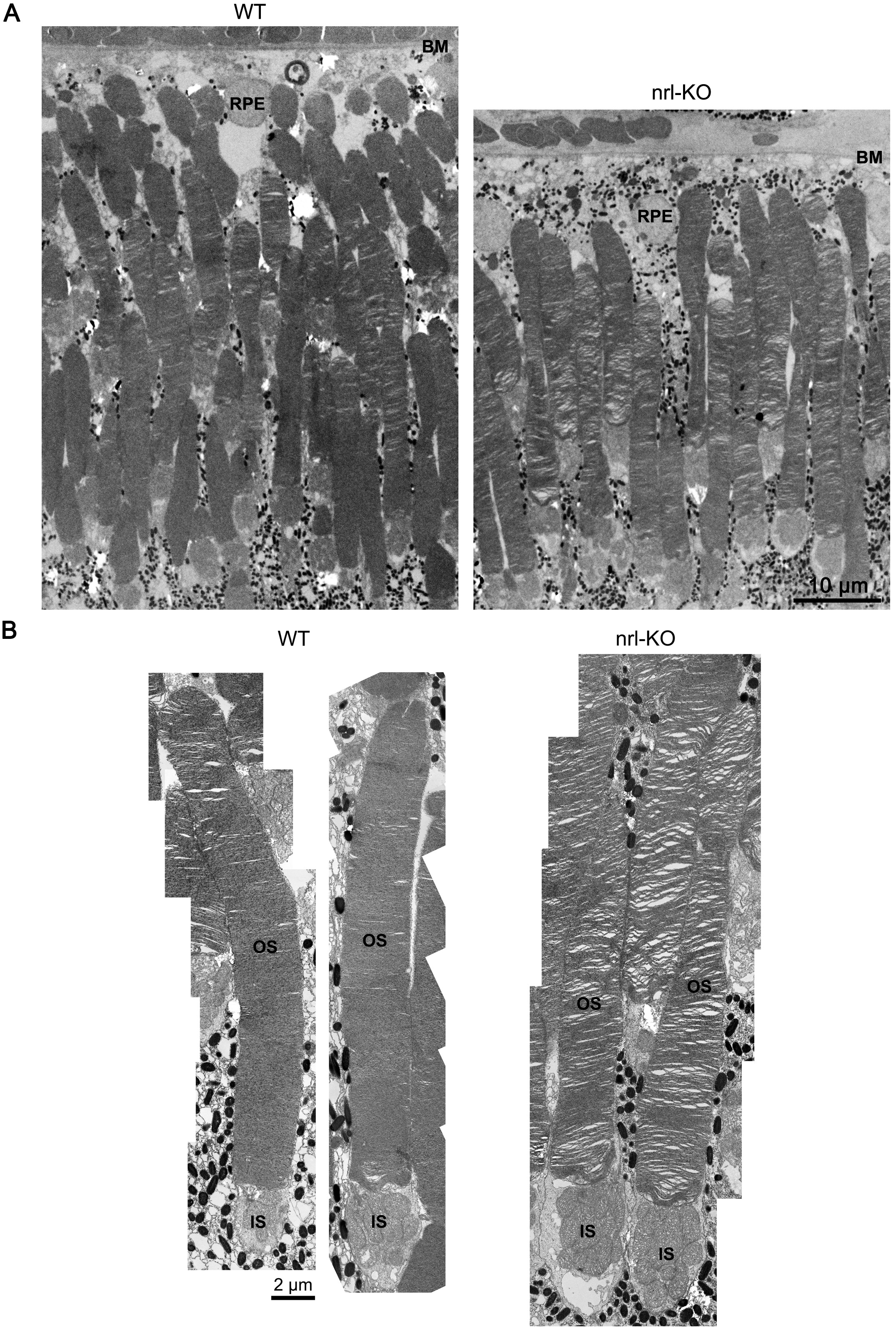

Supplement: S5 Fig — (A) The outer segments of rods are shown in low-magnification TEM image. BM, Bruch’s membrane. RPE, retinal pigment epithelium. Scale bar, 10 μm. (B) Single outer segments of rods are shown in high-magnification TEM images. Compared with the rods in the WT group, most of the rods in the nrl-KO retinas showed a relatively loose membrane disc structure. There was no large difference in the length of outer segments between the WT and nrl-KO rods. OS, outer segment. IS, inner segment. Scale bar, 2 μm. (TIF) [file pgen.1009841.s005.tif]

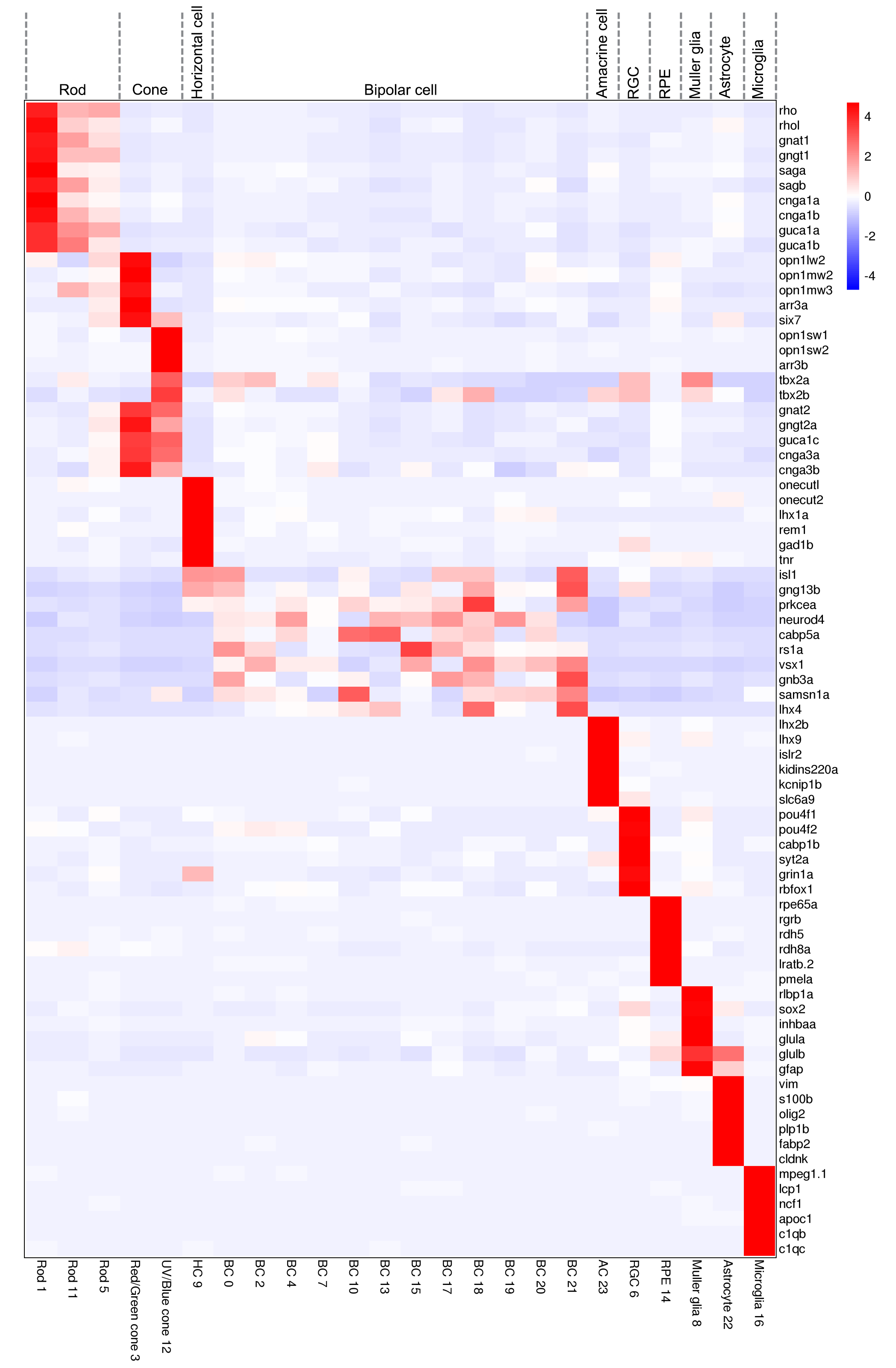

Supplement: S6 Fig — All of the unsupervised cell clusters identified by scRNA-seq were matched to the known retinal cell types. (TIF) [file pgen.1009841.s006.tif]

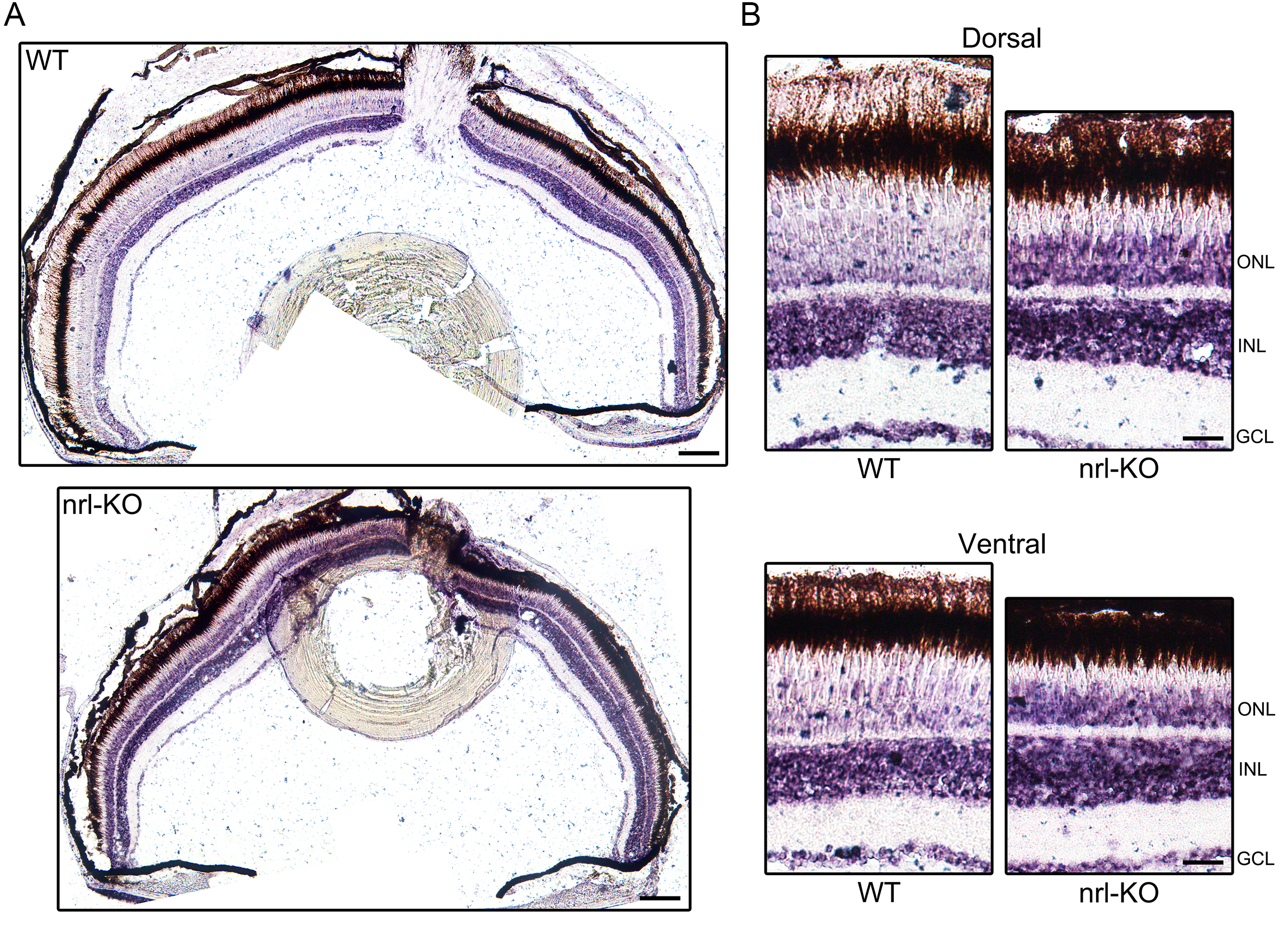

Supplement: S7 Fig — (A) In situ hybridization analysis of hmgn2 on retinal sections from the WT and nrl-KO zebrafish at 3 mpf. Scale bars: 100 μm. (B) Enlarged images of the dorsal and ventral regions of WT and nrl-KO retinas are shown. Scale bars: 25 μm. (TIF) [file pgen.1009841.s007.tif]

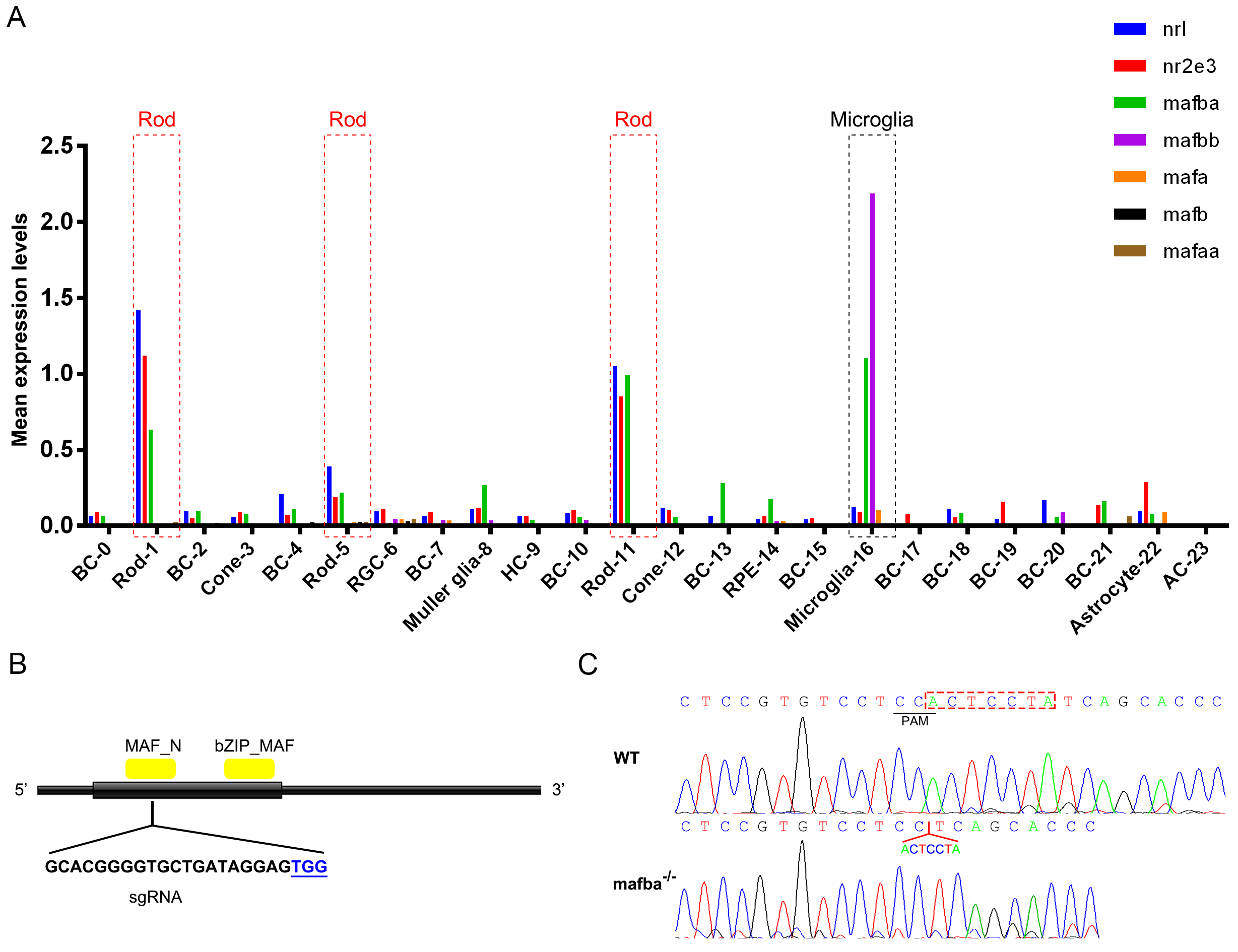

Supplement: S8 Fig — (A) The mean expression levels of nrl, nr2e3, mafba, and other large MAF family genes in each retinal cell type of the zebrafish are shown. Like nrl and nr2e3, the mafba gene (indicated by green bars) was highly expressed in all the three types of rods like nrl and nr2e3. Additionally, mafba was also expressed in the microglia. (B) The protein domains, gene structure, and CRISPR-Cas9 target site of mafba are shown. (C) Validation of the mafba knockout zebrafish carrying the homozygous del7 mutation (c.175_181del7, p.T59Sfs*43) via sequencing. The red box (upper panel) and the red line (lower panel) indicate the deleted 7 bp region in the mafba gene. (TIF) [file pgen.1009841.s008.tif]
