# Supplementary material for: Rod genesis driven by mafba in an nrl knockout zebrafish model with altered photoreceptor composition and progressive retinal degeneration
Source: PLoS Genet. 2022 Mar 4;18(3):e1009841. doi: 10.1371/journal.pgen.1009841 (PMC8926279; doi:10.1371/journal.pgen.1009841)
Supplement: S1 Table — (DOC) [file pgen.1009841.s010.doc]

**S1 Table.** The top 20 marker genes for the three rod clusters identified via scRNA-seq

| Target Cluster | Gene name | Target Cluster mean | Other Cluster mean | Log2FC | Pvalue |
| --- | --- | --- | --- | --- | --- |
| rod-1 | cnga1a | 60.9 | 3.3 | 4.22 | 0.00E+00 |
| rod-1 | cnga1b | 35.8 | 2.8 | 3.67 | 0.00E+00 |
| rod-1 | zgc:162144 | 71.7 | 6.1 | 3.56 | 0.00E+00 |
| rod-1 | saga | 44.1 | 3.8 | 3.55 | 0.00E+00 |
| rod-1 | rhol | 16.2 | 1.4 | 3.53 | 0.00E+00 |
| rod-1 | rho | 1691.3 | 166.4 | 3.35 | 0.00E+00 |
| rod-1 | gnat1 | 118.1 | 11.7 | 3.33 | 0.00E+00 |
| rod-1 | gngt1 | 118.1 | 13.1 | 3.17 | 0.00E+00 |
| rod-1 | sagb | 21.4 | 2.5 | 3.09 | 0.00E+00 |
| rod-1 | pde6gb | 275.9 | 33.4 | 3.05 | 0.00E+00 |
| rod-1 | si:ch211-113d22.2 | 125.0 | 15.4 | 3.02 | 0.00E+00 |
| rod-1 | guca1b | 17.9 | 2.2 | 3.02 | 0.00E+00 |
| rod-1 | unc119.2 | 5.9 | 0.8 | 2.90 | 0.00E+00 |
| rod-1 | guca1a | 12.1 | 1.6 | 2.89 | 0.00E+00 |
| rod-1 | rom1b | 69.5 | 9.7 | 2.84 | 0.00E+00 |
| rod-1 | grk1a | 6.2 | 0.9 | 2.78 | 0.00E+00 |
| rod-1 | nme3 | 6.4 | 1.0 | 2.74 | 0.00E+00 |
| rod-1 | cabp4 | 12.3 | 2.0 | 2.63 | 0.00E+00 |
| rod-1 | rom1a | 7.5 | 1.2 | 2.61 | 0.00E+00 |
| rod-1 | pde6ga | 95.5 | 15.9 | 2.58 | 0.00E+00 |
| rod-5 | ppdpfa | 188.4 | 50.5 | 1.90 | 8.84E-118 |
| rod-5 | mt-nd4l | 9.7 | 2.6 | 1.90 | 1.37E-94 |
| rod-5 | mt-nd5 | 32.8 | 9.1 | 1.84 | 1.75E-168 |
| rod-5 | mt-atp8 | 5.9 | 1.7 | 1.80 | 2.90E-60 |
| rod-5 | ckmt2b | 6.2 | 1.8 | 1.79 | 6.89E-57 |
| rod-5 | mt-atp6 | 477.0 | 154.5 | 1.63 | 3.59E-241 |
| rod-5 | mt-nd2 | 69.0 | 23.5 | 1.56 | 4.39E-181 |
| rod-5 | mt-nd1 | 134.2 | 49.3 | 1.44 | 3.99E-207 |
| rod-5 | mt-nd4 | 123.5 | 45.5 | 1.44 | 6.02E-204 |
| rod-5 | si:dkey-17e16.15 | 5.9 | 2.2 | 1.40 | 7.98E-42 |
| rod-5 | rom1b | 39.5 | 15.1 | 1.38 | 4.71E-88 |
| rod-5 | mt-nd3 | 37.1 | 14.5 | 1.35 | 6.29E-134 |
| rod-5 | mt-co3 | 480.3 | 190.0 | 1.34 | 1.32E-245 |
| rod-5 | mt-co2 | 555.3 | 226.2 | 1.30 | 1.66E-236 |
| rod-5 | rho | 771.5 | 316.7 | 1.28 | 4.52E-101 |
| rod-5 | slc25a3a | 16.1 | 6.7 | 1.27 | 2.62E-61 |
| rod-5 | ckmt2a | 12.5 | 5.3 | 1.24 | 3.58E-46 |
| rod-5 | pde6gb | 134.5 | 56.9 | 1.24 | 3.97E-87 |
| rod-5 | mt-co1 | 254.5 | 108.1 | 1.24 | 3.02E-185 |
| rod-5 | arl3l2 | 10.3 | 4.5 | 1.22 | 4.69E-48 |
| rod-11 | tmsb | 3.9 | 0.1 | 5.07 | 7.20E-286 |
| rod-11 | cxxc5a | 5.3 | 0.2 | 4.47 | 1.36E-141 |
| rod-11 | si:ch211-222l21.1 | 4.1 | 0.3 | 3.61 | 1.26E-97 |
| rod-11 | hmgn2 | 17.9 | 2.2 | 3.02 | 2.48E-40 |
| rod-11 | rcvrna | 3.8 | 0.6 | 2.72 | 1.01E-110 |
| rod-11 | fabp7a | 22.8 | 4.0 | 2.51 | 5.28E-116 |
| rod-11 | kcnv2a | 6.3 | 1.1 | 2.47 | 4.61E-119 |
| rod-11 | tmtops2a | 2.5 | 0.5 | 2.42 | 6.74E-87 |
| rod-11 | kri1 | 4.8 | 0.9 | 2.35 | 1.15E-95 |
| rod-11 | rgs9b | 5.5 | 1.1 | 2.31 | 6.88E-86 |
| rod-11 | hsp90aa1.2 | 16.5 | 3.4 | 2.28 | 1.56E-66 |
| rod-11 | hmgb2b | 13.4 | 2.8 | 2.25 | 1.23E-36 |
| rod-11 | rplp2l | 35.9 | 7.7 | 2.23 | 4.25E-33 |
| rod-11 | eif3ea | 2.8 | 0.6 | 2.16 | 8.99E-65 |
| rod-11 | pde6ga | 101.1 | 23.2 | 2.12 | 1.27E-24 |
| rod-11 | aanat2 | 2.4 | 0.6 | 2.10 | 6.72E-33 |
| rod-11 | pdca | 15.0 | 3.5 | 2.08 | 5.24E-56 |
| rod-11 | rgs9bp | 2.2 | 0.5 | 2.08 | 7.56E-57 |
| rod-11 | prom1b | 2.5 | 0.6 | 2.04 | 1.30E-69 |
| rod-11 | wdcp | 2.4 | 0.6 | 2.03 | 5.36E-55 |
