# Supplementary material for: Rod genesis driven by mafba in an nrl knockout zebrafish model with altered photoreceptor composition and progressive retinal degeneration
Source: PLoS Genet. 2022 Mar 4;18(3):e1009841. doi: 10.1371/journal.pgen.1009841 (PMC8926279; doi:10.1371/journal.pgen.1009841)
Supplement: S2 Table — (DOC) [file pgen.1009841.s011.doc]

**S2 Table.** The antibodies used in this study

| **Antibodies** | **Source** | **Identifier** | **Dilution** |
| --- | --- | --- | --- |
| Anti-zebrafish Rho | Abclonal (custom) | AB_2864372 | 1:500 for WB, 1:200 for IF |
| Anti-zebrafish Opn1mw | Abclonal (custom) | AB_2864373 | 1:50 for IF |
| Anti-α-Tubulin | Millipore | AB_10807281 | 1:3000 for WB |
| Anti-GNAT1 | Abclonal | AB_2619670 | 1:500 for WB, 1:50 for IF |
| Anti-GNAT2 (Zebrafish) | MBL | AB_2864374 | 1:500 for WB |
| Anti-GNB1 | Abgent | AB_10664825 | 1:1000 for WB |
| Anti-GNB3 | Proteintech | AB_2263264 | 1:1000 for WB |
| Anti-PCNA | Proteintech | AB_2160330 | 1:200 for IF |
| Anti-TJP1 (ZO-1) | Abclonal | AB_2758549 | 1:100 for IF |
| Anti-GFAP | GeneTex | AB_2814877 | 1:100 for IF |
| Goat anti-Rabbit IgG (H+L) Secondary Antibody, HRP | Thermo Fisher Scientific | AB_228341 | 1:20000 for WB |
| Goat anti-Mouse IgG (H+L) Secondary Antibody, HRP | Thermo Fisher Scientific | AB_228307 | 1:20000 for WB |
| Goat anti-Rabbit IgG (H+L) Secondary Antibody, Alexa Fluor 488 | Thermo Fisher Scientific | AB_2534114 | 1:1000 for IF |
| Goat anti-Rabbit IgG (H+L) Secondary Antibody, Alexa Fluor 594 | Thermo Fisher Scientific | AB_2534079 | 1:1000 for IF |
| Goat anti-Mouse IgG (H+L) Secondary Antibody, Alexa Fluor 488 | Thermo Fisher Scientific | AB_2534069 | 1:1000 for IF |
| Goat anti-Mouse IgG (H+L) Secondary Antibody, Alexa Fluor 594 | Thermo Fisher Scientific | AB_2534073 | 1:1000 for IF |
