# Supplementary material for: Rod genesis driven by mafba in an nrl knockout zebrafish model with altered photoreceptor composition and progressive retinal degeneration
Source: PLoS Genet. 2022 Mar 4;18(3):e1009841. doi: 10.1371/journal.pgen.1009841 (PMC8926279; doi:10.1371/journal.pgen.1009841)
Supplement: S3 Table — (DOC) [file pgen.1009841.s012.doc]

**S3 Table.** The primers used for qPCR in this study

| **Genes** | **Forward** | **Reverse** |
| --- | --- | --- |
| *nrl* | GAACAGACATTTGCGTGGGG | TCGGGCTTTGTACGTGTCTC |
| *rho* | CGCACACCCCTCAACTACAT | GCGACTTTAGCCCCATCTCA |
| *opn1sw1* | tcccgagggtcttggcactg | ctgccttctgggtggactctgac |
| *opn1sw2* | TTTGCGGTTCCTTTCAGCAC | GGTTGCCAGTCTCAGGTCAA |
| *opn1lw1* | TAAGTGACTACAGGTTTGGGCT | GGGGCAATGTGGTAATTGGG |
| *opn1lw2* | AGAGCGCCACCATCTACAAC | AGTCCAGTTCTTCCCTCTTGT |
| *opn1mw1* | ACTTTGGTGGTTACAGCCCA | CTGACCTCCAAGTGTTGCGA |
| *opn1mw2* | TGGCTGGTCCCGATACATCC | TGTGCAAACGAGGCTTCCATAA |
| *opn1mw3* | CGCTGGGATTGGATTTACATGG | GGCAGCAGAACATGTAGAGGAC |
| *opn1mw4* | TCACGCTTTCGCAGGATG | TGTGCAAACAAGTCGCCCAT |
| *nr2e3* | TCAAACCAGAAACACGAGGACTT | GGTGTGTTCCCGATTGTTCTCT |
| *mafba* | AACGCGAAGAACACGGTAGA | CCTCACCATACCAGACGCAA |
