# Supplementary material for: Rod genesis driven by mafba in an nrl knockout zebrafish model with altered photoreceptor composition and progressive retinal degeneration
Source: PLoS Genet. 2022 Mar 4;18(3):e1009841. doi: 10.1371/journal.pgen.1009841 (PMC8926279; doi:10.1371/journal.pgen.1009841)
Supplement: Attachment — Submitted filename: PLosGenetics-Review.docx [file pgen.1009841.s018.docx]

Reviewer: B. E. Knox

Manuscript: PGENETICS-D-21-01260. Liu et al., Rod-genesis driven by mafba in an nrl knockout zebrafish model with altered photoreceptor compositions and progressive retinal degeneration

The genetic regulatory networks that control retinal differentiation have been deeply studied in many organisms, but there remain many unanswered questions. An emergent aspect of this problem is the conservation and divergence of gene function during evolution. It is clear that while there are many similarities between lower vertebrates and mammals in the steps that lead to the adult retina and related control of gene expression, there has been complicated and not well-understood alterations in the GRNs between fish, birds and mammals. Nrl has attracted a great deal of attention because of its central role in mammalian rod photoreceptor differentiation. In mice, Nrl is required for the formation of rods and expression of rod-specific genes, and is entirely dedicated to this role. In lower vertebrates, Nrl has more varied functions, but it has not been studied by genetic ablations until recently in zebrafish (Reference 59, Oel et al., 2020,). Oel et al. show that in larva, Nrl KO animals lacked rods and had more S-cones, but that rods (and rhodopsin expression) were abundant in adult fish. The manuscript under review covers similar ground.

Liu et al. report on the phenotype of Nrl knock-out zebrafish using a wide variety of techniques. The histological phenotype of the Liu et al. Nrl KO are largely confirmatory of the Oel et al. work. Both groups show a large reduction in rods in young fish and a partial “recovery” of rod numbers in adults. Both groups also report on an increase in cones concomitant with rod loss. However, there are important discrepancies. Oel et al. find an increase in S-cones (immunolabeled with 10C9.1; ZFIN ID: ZDB-ATB-140728-2, rat anti-trout UV cone opsin that recognizes the N-terminus) that occurs with rod loss in larval fish, while they find no differences in cone or rod abundances in adults. Liu et al. find an increase in in M-cone number and changes in cellular morphology in older fish, and accompanying increases in MW opsin RNA levels. What are the reasons for this? Unfortunately, Liu et al. neither acknowledge (except in one sentence, 362-364) nor discuss the results of Oel et al., which in large part has already covered the major points in Liu et al. This is a major deficiency that must be addressed in a comprehensive way.

There are a number of very findings in this paper (Liu et al.) that this reviewer finds very important and a significant advance for the field, starting with Figure 5:

1) The cluster analysis of single-cell sequencing identifies 3 subtypes of rods, only one of which (Rod 1) appears to be lost in the Nrl KO fish. The features that distinguish Rod 5 and Rod 11 from the Nrl-sensitive cluster is not well presented, the reader will have to dig into the data themselves to understand the signatures. In addition, Figure 5E is not convincing-a higher, confocal series is needed to assess ROS-GCOS staining.

2) ­The role of MafBa in the formation of rods in the adult is a fascinating observation, and should be perhaps the main point of the paper (especially given Oel et al.). This deserves a more thoughtful presentation, and a discussion to put this fact into (evolutionary) perspective. The gene expression data in Fig. 6 does not support their conclusion that MafBa shows “the closest relationship with nrl and nr2e3 (l. 281-283). I think the MafBa KO does a better job, but this point needs to addressed. As is, the authors do a disservice to their results and miss a chance to frame the next stage of investigation for this field.

3) The authors find indications of reactivity with age in Nrl KO retina (Figure 7). This is an interesting observation. But the presentation and Figure 7 are confusing. The retina is not really degenerating since it seems to be stable in older fish, the RPE is disordered but does not undergo permanent loss (?). It appears that the retina is undergoing some glial reactivity (Figure 7D) but does it recover, stabilize or ? The manuscript is very confusing for this section, and it does not seem correct to talk about degeneration in this model.

Finally, the paper requires substantial editing for language, more precise meaning (there are too many instances to enumerate) and typos (eg. p. 11 line 199 reference to Fig. 3C is incorrect, should be Fig. 3B and in Figure 6A , the reference to mafba is listed as mafb). There are no figure legends for the supplementary figures, which makes them very difficult to understand.
