# Supplementary material for: Rod genesis driven by mafba in an nrl knockout zebrafish model with altered photoreceptor composition and progressive retinal degeneration
Source: PLoS Genet. 2022 Mar 4;18(3):e1009841. doi: 10.1371/journal.pgen.1009841 (PMC8926279; doi:10.1371/journal.pgen.1009841)
Supplement: Attachment — Submitted filename: Response to reviewers.docx [file pgen.1009841.s019.docx]

Reviewer: B. E. Knox

Manuscript: PGENETICS-D-21-01260. Liu et al., Rod-genesis driven by mafba in an nrl knockout zebrafish model with altered photoreceptor compositions and progressive retinal degeneration

The genetic regulatory networks that control retinal differentiation have been deeply studied in many organisms, but there remain many unanswered questions. An emergent aspect of this problem is the conservation and divergence of gene function during evolution. It is clear that while there are many similarities between lower vertebrates and mammals in the steps that lead to the adult retina and related control of gene expression, there has been complicated and not well-understood alterations in the GRNs between fish, birds and mammals. Nrl has attracted a great deal of attention because of its central role in mammalian rod photoreceptor differentiation. In mice, Nrl is required for the formation of rods and expression of rod-specific genes, and is entirely dedicated to this role. In lower vertebrates, Nrl has more varied functions, but it has not been studied by genetic ablations until recently in zebrafish (Reference 59, Oel et al., 2020,). Oel et al. show that in larva, Nrl KO animals lacked rods and had more S-cones, but that rods (and rhodopsin expression) were abundant in adult fish. The manuscript under review covers similar ground.

Liu et al. report on the phenotype of Nrl knock-out zebrafish using a wide variety of techniques. The histological phenotype of the Liu et al. Nrl KO are largely confirmatory of the Oel et al. work. Both groups show a large reduction in rods in young fish and a partial “recovery” of rod numbers in adults. Both groups also report on an increase in cones concomitant with rod loss. However, there are important discrepancies. Oel et al. find an increase in S-cones (immunolabeled with 10C9.1; ZFIN ID: ZDB-ATB-140728-2, rat anti-trout UV cone opsin that recognizes the N-terminus) that occurs with rod loss in larval fish, while they find no differences in cone or rod abundances in adults. Liu et al. find an increase in in M-cone number and changes in cellular morphology in older fish, and accompanying increases in MW opsin RNA levels. What are the reasons for this? Unfortunately, Liu et al. neither acknowledge (except in one sentence, 362-364) nor discuss the results of Oel et al., which in large part has already covered the major points in Liu et al. This is a major deficiency that must be addressed in a comprehensive way.

Response:

We appreciate the reviewers for their careful readings and valuable comments. Most of the experiments mentioned in this study were finished before 2020, and the manuscript was prepared in the first half of 2020. At that time, we have no idea about the results of Oel *et al.* (reference 59, published at 18 December 2020). This is one major reason that their results were rarely discussed in our original manuscript. We fully understand and respect the reviewer’s comments. A comparison of the retinal phenotypes of the two *nrl* knockout zebrafish models were included in the revised manuscript (page 21-22, line 388-400). The main contents are briefly described as follows.

(1) The two studies both demonstrated that *nrl* is necessary for the development of rod photoreceptors at the embryonic stage but not the adult stage in zebrafish. In addition, our study showed that in adult *nrl* knockout zebrafish, the number of rods was reduced to a certain extent, as validated by multiple types of experiments (such as immunofluorescence, qPCR, western blot, RNA-seq, scRNA-seq) at both the cellular and molecular levels.

(2) Oel *et al* found an increase (~30%) of UV cones in the *nrl* mutants at 4 dpf by immunostaining of whole-mount retinas. However, the over-production of UV cones could not be detected in adults. In our study, the mRNA levels of UV opsin (*opn1sw1*) were unchanged at 2 mpf and 5 mpf in the *nrl* mutants, as detected by qPCR and RNA-seq. We also imaged the outer segments of UV cones by immunostaining using a customized antibody (Abclonal Technology, Wuhan, China) against the zebrafish UV opsin on retinal sections of WT and nrl-KO zebrafish at 14 dpf (please see the figure below). No apparent increase of UV cones was observed. Lineage tracing experiments (reference 29, [PMID: 27326930](https://pubmed.ncbi.nlm.nih.gov/27326930/)) have shown that zebrafish rods are unlikely derived from UV- and blue-cone precursors (corresponding to S-cone precursors in mice). Besides, our previous study (reference 30, [PMID: 30684641](https://pubmed.ncbi.nlm.nih.gov/30684641/)) has shown that knockout out of *nr2e3* in zebrafish doesn’t cause the increase of UV cones, too. Therefore, we did not further monitor the changes of UV cones in subsequent experiments. Two reasons may explain the difference results about UV cones in the two studies: (a) The increase of UV cones is moderate and only present in a short time window; (b) Immunostaining on retinal sections may not be sensitive enough to reveal the slightly increased UV cones.

(3) We found that the increase of green cones (M-cones) in *nrl* knockout zebrafish is an age-related process starting at 2~3 mpf. At 13 mpf, the increase of green-cone outer segments became quite evident (the new Fig 6D), as detected by immunostaining on retinal sections using a customized antibody (Abclonal Technology, Wuhan, China) against the zebrafish green-cone opsin (Opn1mw1). Through western blot, qPCR, and RNA-seq, we also demonstrated the over-growth of green cones in adult *nrl* mutants (from 2 mpf to 5 mpf) at the molecular level. We speculate that the age of the zebrafish tested, which was not mentioned in the *iScience* paper (reference 59, [PMID: 33299975](https://pubmed.ncbi.nlm.nih.gov/33299975/)), may be the key reason that causes the inconsistent description about the green cones in the two studies.

There are a number of very findings in this paper (Liu et al.) that this reviewer finds very important and a significant advance for the field, starting with Figure 5:

1) The cluster analysis of single-cell sequencing identifies 3 subtypes of rods, only one of which (Rod 1) appears to be lost in the Nrl KO fish. The features that distinguish Rod 5 and Rod 11 from the Nrl-sensitive cluster is not well presented, the reader will have to dig into the data themselves to understand the signatures. In addition, Figure 5E is not convincing-a higher, confocal series is needed to assess ROS-GCOS staining.

Response:

We have made a new supplementary table (the new S1 Table) to show the top 20 marker genes for the three clusters of rods. As these genes have not been verified by other experiments, we didn't list them separately in the original manuscript. The old Fig 5E has been changed with higher resolution images, which were synthesized by confocal series, in the revised manuscript (the new Fig 7E).

2) The role of MafBa in the formation of rods in the adult is a fascinating observation, and should be perhaps the main point of the paper (especially given Oel et al.). This deserves a more thoughtful presentation, and a discussion to put this fact into (evolutionary) perspective. The gene expression data in Fig. 6 does not support their conclusion that MafBa shows “the closest relationship with nrl and nr2e3 (l. 281-283). I think the MafBa KO does a better job, but this point needs to addressed. As is, the authors do a disservice to their results and miss a chance to frame the next stage of investigation for this field.

Response:

Thanks for the review’s suggestions. We have added a new figure (the new Fig 9) to show the gene collinearity of *mafba* among species in different evolutionary positions. Our analysis showed that *mafba* and *mafbb* have arisen by the teleost fish-specific duplication of the ancestral gene *mafb*. Their functions may gradually diverge during evolution, which causes the unique role of *mafba* in rod development in modern fishes such as zebrafish, medaka, and so on.

The old Fig 6A (the new Fig 8A) showed the relative expression levels (row-normalized) of photoreceptor development related genes and large MAF genes in each type of retinal cells. The expression pattern of *mafba* (specifically expressed in rod 1, rod 11, and microglia) is more similar to *nrl* and *nr2e3* (see the first three rows in the new Fig 8A) than other large MAF genes. The old Fig S7A (the new Fig S8A) showed the mean expression levels of *nrl*, *nr2e3*, and large MAF genes in each retinal cell types. Except for *mafba* (indicated by green bars in the new Fig S8A), which showed similar expression levels to *nrl* and *nr2e3* in rods, other large MAF genes (*mafbb*, *mafa*, *mafb*, *mafaa*) were almost not expressed in rod cells. These results suggested that *mafba* (but not other large MAF genes) likely play a role in rods. We have modified the description about this problem in the revised manuscript (page 17, line 295-296). We have generated the *mafba* knockout zebrafish (the new Fig S8B and S8C) and crossed it with nrl-KO zebrafish. Double knockout of *nrl* and *mafba* indeed eliminated the residual rods observed in the *nrl* single knockout zebrafish (the new Fig 8B).

3) The authors find indications of reactivity with age in Nrl KO retina (Figure 7). This is an interesting observation. But the presentation and Figure 7 are confusing. The retina is not really degenerating since it seems to be stable in older fish, the RPE is disordered but does not undergo permanent loss (?). It appears that the retina is undergoing some glial reactivity (Figure 7D) but does it recover, stabilize or ? The manuscript is very confusing for this section, and it does not seem correct to talk about degeneration in this model.

Response:

We have detected the apoptosis of retinal cells and activation of Müller glia in nrl-KO zebrafish, both of which are hallmark features of retinal degeneration. However, as mentioned by the reviewer, the degeneration process was quite slow and stable for a long period of time. The activation of retinal regeneration (indicated by more Pcna-positive cells in ONL, see the new Fig 10E) may be responsible for this situation. Abnormal morphology of RPE cells is also a manifestation of retinal degeneration. The enlarged RPE cells may represent a compensation effect to the loss of RPE cells (reference [PMID: 19850870](https://pubmed.ncbi.nlm.nih.gov/19850870/)). Generally, activation of glial cells in progressively degenerating retinas is thought to be a kind of inflammatory response and will last for a long time.

At the beginning of the study, one of our purposes is to establish a zebrafish model for enhanced S-cone syndrome (ESCS), which is caused by mutations in *NR2E3* and *NRL* in humans. In ESCS patients, the loss of rods and over-production of S-cones cause severe retinal degeneration. The pathological mechanism of ESCS remains unclear. However, the surprising phenotypes shown in this study suggested that the nrl-KO zebrafish is not an appropriate disease model for ESCS. Even so, the remodeling of photoreceptor compositions in nrl-KO retinas could still cause a slowly progressing retinal degeneration, which might provide some valuable clues for further studies.

Finally, the paper requires substantial editing for language, more precise meaning (there are too many instances to enumerate) and typos (eg. p. 11 line 199 reference to Fig. 3C is incorrect, should be Fig. 3B and in Figure 6A , the reference to mafba is listed as mafb). There are no figure legends for the supplementary figures, which makes them very difficult to understand.

Response:

We really apologize for the clerical errors and shortcomings in English writing. The manuscript has been checked for several times to fix the problems and proofread by two native English speakers (Dr. James Reilly and Dr. Xinhua Shu in the Glasgow Caledonian University, Glasgow, UK). The legends for supplementary figures were included in a separate supplementary document in the original manuscript. We have put all the supplementary figures and legends in a single document in the revised manuscript.

Reviewer #2: PGENETICS-D-21-01260 Liu et. al “Rod-genesis driven by mafba in an nrl knockout zebrafish model with altered photoreceptor compositions and progressive retinal degeneration”

This manuscript describes the identification of a gene, mafba, as a novel regulator for nrl-independent rod formation in zebrafish. The authors generated an nrl knockout zebrafish using CRISPR-Cas9, and saw a reduction but not total elimination of rods along with an overgrowth of green cones. There are major and minor comments to be addressed prior to publication. It is strongly recommended that the manuscript be restructured such the main thrust of the data are in the main body of the text and the supplemental data for incremental data for ease of reading.

Response: We thank the reviewer’s useful suggestions. We have restructured the figures and some text in the main body in the revised manuscript.

Major comments:

Abstract/Author Summary

The authors state that “abnormal gene expression caused progressive retinal degeneration and subsequent regeneration in nrl knockout zebrafish.” Since zebrafish are able to regenerate damaged retinas through the Müller glia-derived progenitors (PMCID: PMC2948409), could these be the source of the second wave of rod genesis they mention earlier in the abstract? This is not addressed in the text. Since this is a viable option for data seen, this needs to be addressed and considered.

Response:

We fully agree with the reviewer’s consideration. Previous studies have shown that there may be three sources of rods in zebrafish retinas (reference 25, [PMID: 27281280](https://pubmed.ncbi.nlm.nih.gov/27281280/)): (a) retinal progenitor cells that produce all types of retinal cells at the embryonic stage; (b) Müller glia-derived unipotent rod progenitors that continuously generate rods throughout the lifetime; (c) injury-induced Müller glia-derived multipotent progenitors that produce all major retinal cell types. The latter two both originate from Müller glial cells, but have different regulatory mechanisms and outcomes. Due to the temporal and spatial similarities, it’s reasonable to speculate that the second wave of rod genesis, which is *nrl*-independent, is likely the “b” type of production of rods from Müller glia-derived unipotent rod progenitors. Besides, we found that the regeneration of rods (“c” type) may also be *nrl*-independent (see the new Fig 8). In other words, *nrl* is likely not required for the generation of rods from Müller glial cells. This part has been discussed in the original manuscript and further improved in the revised manuscript (page 21, line 379-387).

The authors state that the importance of these findings in zebrafish “highlight the conserved and species-specific regulatory mechanisms of photoreceptor development and maintenance” however the difference between zebrafish photoreceptor development and mammalian photoreceptor development implies they are not a great model for human disease mechanism studies. This is especially true since these results are in contrast with the Nrl knockout mice. From this point, it would be reasonable if the authors restructure their paper such that they are making the point that zebrafish are not a good model for human disease, and they have discovered data underpinning zebrafish development which may not be applicable to human retinal development. This reviewer realizes this is not an exciting or is perhaps controversial, but the data presented here do not correlate with mouse development, and therefore may be different in humans as well. Either way, they do not support the statement above.

Response:

Whether mice or zebrafish, they both show conserved and species-specific features of retinas when compared with humans. What’s more important is to exert the advantages and avoid the disadvantages of these animal models in research. In this case, the retinal phenotypes of nrl-KO zebrafish indeed do not support it as an appropriate model of enhanced S-cone syndrome (ESCS). However, numerous studies have shown that zebrafish is an useful animal model for inherited retinal degenerations, especially for those genes involved in phototransduction, visual cycle, protein trafficking, splicing, and so on. We still believe in the value of zebrafish as a model in the field of retinal degeneration. Nevertheless, some statements may be inappropriate as suggested by the reviewer. We have modified them in the revised manuscript.

“NRL is the most important gene for the occurrence and function of rod cells in mice and humans” – This sort of declarative statement can be refuted and as such care should be taken in overstatements like this throughout the text.

Response: Thanks for the reviewer’s suggestion. We have modified this sentence and other similar problems in the revised manuscript.

Line 36: “over-grown of” should be replaced with “over-growth of” or “compensation by”

Line 60-61: “breaking the function”? this phrase is confusing

Response: Thanks for the reviewer’s suggestions. We have fixed the problems in the revised manuscript.

Results

Figure 1

Zebrafish Nrl antibodies couldn’t be used for western or IF. This in concert with an increase in mRNA does not result in confidence that they are getting a true knockout of nrl.

Response:

We are very confident about the knockout of *nrl* for the following reasons. (1) The CRISPR-Cas9 target site is very close to the target site used in Oel *et al.*’s study (reference 59). They are expected to result in the same consequence. (2) Both studies have shown that there are no other transcripts and alternative splicing events for zebrafish *nrl* gene. The frameshift mutation (c.230_237del8, p.P77Hfs*4) was expected to cause loss of the two key domains of Nrl protein. (3) The increased mRNA levels of *nrl* were observed in both studies (Fig 1C in this study and Fig 5 in Oel *et al.*’s study), as detected by qPCR and *in situ* hybridization. This may be due to a negative feedback regulation of *nrl* gene expression. The reason why our customized Nrl antibodies don't work may be due to the immunogen (30-286 aa of zebrafish Nrl) we used. Oel *et al.* have generated a workable zebrafish Nrl antibody using the C-terminal 200 aa as immunogen in their study.

Figure S1

Why are they comparing different ages 1 mpf and 2 mpf WT and KO, respectively?

They have rod nuclei (presumably) in H&E stained sections at 2 mpf in KO but decrease in thickness of PR+RPE layer – is this due to loss of outer segments?

Response:

Fig S1 is one of our early results that shows the overall retinal structure of nrl-KO zebrafish at 1 mpf and 2 mpf. From these results, we realized for the first time that rods were not totally eliminated by *nrl* knockout in zebrafish. After that, we used a variety of methods to explore the unexpected phenotype and the underlying molecular mechanism. Considering the developmental process of rods and the apoptosis detection results, we think that the decrease in thickness of PR+RPE layer is likely due to the fewer production of rods in nrl-KO zebrafish.

Figure 2/S2

The authors state that thickness of rod OS and number of rod nuclei is significantly reduced – but this is not quantified anywhere in figure2 or S2. Likewise, it is stated on line 163, “we didn’t observe the fluorescence of rods until 7 dpf in KP zebrafish”. Please see figure and legend S4A/B. There are some rods; little, but some.

Response:

The exact time point when the second wave of rod cells begin to appear highly depends on the growth conditions of zebrafish larvae. A high breeding density will greatly delay the second wave of rod genesis, but has little effect on the first wave of rod genesis (beginning at about 3dpf). Some nrl-KO larvae may have very few rods at 7 dpf. We have modified the inappropriate description in the revised manuscript. The quantitative data have been added (the new Fig S3A).

What is the difference in Rho/anti-Rho labeling? Why do they go back and forth between red and green? This is confusing.

Response:

We apologize for the confusing presentation. There is no difference between the two labels. All images were acquired by immunofluorescence using the anti-zebrafish Rho antibody. The red and green colors represent the different fluorescent secondary antibodies used in the experiments. This figure has been modified in the revised manuscript (the new Fig S2).

Figures 3 & S3

They state that density of rod OS significantly reduce in nrl-KO zebrafish and show staining in S3 but it is not quantified anywhere in S3.

Same with the number of rod numbers “obviously reduce” on page 10. They never quantify.

Response: The quantitative data have been added in the revised manuscript (the new Fig S3B).

In the text, they talk about mRNA levels of cone specific genes being significantly changed and reference 3C, but this is not what 3C shows. I assume they mean 3B.

Response: We apologize for the careless mistake. This problem has been corrected in the revised manuscript.

Figures 4 & S4

A – it is unclear if these are separate animals/in triplicate or duplicate/ or if they’re different time points quantified in B

Response:

Each column of immunoblot image shows a set of representative results in an independent experiment. For each age, at least three independent repeats were carried out. Each lane in the image represents a sample from a separate animal.

B – It is difficult to tell which bars go with what WT in the legend. Consider separating by age and comparing WT with KO. S4B should be in the manuscript, not supplemental.

Response: Thanks for the reviewer’s suggestions. The old Fig 4B has been remade in the revised manuscript (the new Fig 6B). All WT samples were labeled with blue color, and all nrl-KO samples were labeled with red color. The data of different time points have been arranged from 14 dpf to 3 mpf in a left-to-right mode and indicated by different fill patterns.

Additionally, the authors state "the fluorescence of every single rod was also significantly lower than WT zebrafish." Is this quantified? It needs to be in order to state this. WT is oversaturated in the image; what do the data look like in sub-saturating conditions?

Response:

Fluorescence signals in the old Fig S4C (the new Fig 4B) were not quantified because it’s very difficult to identify the boundaries of rod cells in these images. This conclusion was based on the observation of our eyes. We have deleted this sentence in the revised manuscript. The images of WT and nrl-KO zebrafish were acquired under the same parameters. In order to see the signals clearly in the nrl-KO group, the cell bodies of WT rods had to be overexposed. The inner and outer segments of WT rods were not oversaturated.

Line 174/5 states that rods were absent in marginal zones, yet this reviewer sees rods. Indeed, the authors say in the figure legend for S4 that "arrows indicate retinal regions without rods." I don't see that; when I increase the size of the figure in C, I do see what appear to be rods.

Response: We have modified the labels and descriptions related to the old Fig S4 (the new Fig 4B). Rods were specifically labeled with EGFP. Compared with WT zebrafish, the regions nearby ciliary marginal zone (labeled with boxes in the new Fig 4B) showed no or little fluorescence signals in nrl-KO zebrafish at 1 mpf.

The authors state that there is limited distribution and decreased expression of rod opsin on retinal sections of KO at 1 mpg. Is this due to transgene? Transmission electron microscopy should be performed to determine the ultrastructure of the OS in the KO animals. In this case, the OS should be shorter and thinner with a decrease in rhodopsin expression. Additionally, dot blots are warranted to quantitatively show a decrease in rhodopsin expression.

Response:

The rho:EGFP transgene zebrafish is widely used in many studies. The promoter (~1 kb upstream the initiation codon) contains a conserved binding site of Nrl. It’s reasonable that the expression of *rho* is reduced in *nrl* knockout zebrafish. The limited distribution of rods in the retinas of young nrl-KO zebrafish is likely due to the lack or delay of rod production in those regions. Dot blot and transmission electron microscopy were performed in WT and nrl-KO zebrafish at 9 mpf (the new Fig S4 and S5). The dot blot result again demonstrated that the protein levels of rhodopsin were decreased in nrl-KO retinas. There was no large difference in the length of outer segments between WT and nrl-KO rods as revealed by TEM. However, the membrane discs in most of the nrl-KO rods seemingly did not stack as tightly as those in WT rods.

Figure 5

The authors show that mafba mRNA levels are decreased at 2, and 5 months in the nrl KO. If it is compensatory or involved in the “nrl-independent” pathway, why would the mRNA levels be lower?

Response:

If there is a genetic compensatory effect, the expression of *mafba* should be up-regulated. However, this possibility was ruled out by our experimental results (the new Fig 8C), suggesting that *mafba* may play a constitute role in the development of zebrafish rods. We guess that the reduction of rods may lead to the decrease of *mafba* mRNA levels, as *mafba* is expressed in all three types of rods according to our scRNA-seq data (the new Fig S8A). The exact reason remains unclear.

Figure 6

The authors make the comment that mafba + nrl double knockout eliminates rods (nrl KO alone does not and mafba KO alone dies at 9 dpf). Since it is a global KO of mafba and nrl, could it be that they are affecting the ability of new progenitors being formed from Müller glia etc. Double knockout with a gene that is lethal at 9 dpf could have many off target issues.

Response:

We do not exclude the possibility. Whether the double knockout of *nrl* and *mafba* inhibits the formation of progenitors from Müller glia or the differentiation of Müller glia-derived progenitors to rods needs a series of follow-up experiments to answer. The *mafba* gene shows extensive functions in many tissues. There may be indirect effects. Cell-type-specific knockout zebrafish lines are needed to address these concerns. Unfortunately, we are unable to solve this problem at the moment.
