# Supplementary material for: Rod genesis driven by mafba in an nrl knockout zebrafish model with altered photoreceptor composition and progressive retinal degeneration
Source: PLoS Genet. 2022 Mar 4;18(3):e1009841. doi: 10.1371/journal.pgen.1009841 (PMC8926279; doi:10.1371/journal.pgen.1009841)
Supplement: Attachment — Submitted filename: Response to reviewers.docx [file pgen.1009841.s020.docx]

Reviewer #1: The authors have addressed my major concerns in this revised manuscript. There are two points that I believe should be addressed. First, the authors have expanded their comparison of the previous (overlapping work) of Oel et al. on p. 21-22. However, I feel the text included in the response to the reviewers is more direct and compelling than that in the paper itself. In fact, I suggest the authors incorporate the response directly into the paper. I also think a few sentences in the introduction to signal that there has been similar work published would help the reader and put both studies in perspective. But this may be a matter of taste. Second, the writing has improved, but it still suffers from significant language problems. I recommend that the paper be edited once more, I believe it will dramatically improve the impact.

Response: Thanks a lot for the reviewers’ positive comments. We accept the reviewer's two new suggestions. The contents about the comparison of the two studies have been integrated into the discussion part (line 388-407 in the revised manuscript). The main body of the manuscript has been sent to a professional company (https://wordvice.com/) for English editing services. A screenshot of the editing certificate is shown below.

Reviewer #2: PGENETICS-D-21-01260

This is a much-improved revision of a manuscript describing the identification of mafba as a novel regulator for nrl-independent rod formation in zebrafish. The authors addressed the major and minor concerns posed by the two reviewers. With minor edits, this reviewer agrees this paper should be published in PLOS Genetics.

Response: We appreciate the reviewers’ positive comments. The minor problems suggested by the reviewer have been solved in the revised manuscript.

Minor edits include:

• Lines 181-182: Most likely, the separation between the disks is due to artifacts of transmission electron microscopy. The last sentence implies nrl is directly involved in disk integrity during outer segment turnover. To state this, even with the qualifier “seemingly”, one would need to do cryoelectron tomography to unveil the disk ultrastructure with that level of precision. To avoid having to do this, I would consider deleting this sentence.

Response: Thanks for the reviewer’s suggestion. We have deleted this sentence in the revised manuscript.

• Line 396: change “What’ more important” with “Importantly”

Response: The phrase has been changed in the revised manuscript

• Line 399: change “tissular” with “tissue”

Response: The word has been changed in the revised manuscript.

• Line 432: this sentence should end with “… and retinal degeneration and regeneration in zebrafish.”

Response: The words “in zebrafish” have been added in the sentence in the revised manuscript.
